# Multielectrode catheter-based pulsed field ablation of persistent and long-standing persistent atrial fibrillation

**DOI:** 10.1093/europace/euae246

**Published:** 2024-10-01

**Authors:** Domenico G Della Rocca, Antonio Sorgente, Luigi Pannone, María Cespón-Fernández, Giampaolo Vetta, Alexandre Almorad, Gezim Bala, Alvise Del Monte, Erwin Ströker, Juan Sieira, Ioannis Doundoulakis, Sahar Mouram, Charles Audiat, Cinzia Monaco, Sanghamitra Mohanty, Roberto Scacciavillani, Lorenzo Marcon, Kazutaka Nakasone, Wael Zaher, Ingrid Overeinder, Serge Boveda, Mark La Meir, Andrea Natale, Andrea Sarkozy, Carlo de Asmundis, Gian-Battista Chierchia

**Affiliations:** Heart Rhythm Management Centre, Postgraduate Program in Cardiac Electrophysiology and Pacing, Universitair Ziekenhuis Brussel-Vrije Universiteit Brussel, European Reference Networks Guard-Heart, Av. du Laerbeek 101, 1090 Jette, Brussels, Belgium; Texas Cardiac Arrhythmia Institute, St. David's Medical Center, 3000 N Interstate Hwy 35, Austin, TX 78705, USA; Heart Rhythm Management Centre, Postgraduate Program in Cardiac Electrophysiology and Pacing, Universitair Ziekenhuis Brussel-Vrije Universiteit Brussel, European Reference Networks Guard-Heart, Av. du Laerbeek 101, 1090 Jette, Brussels, Belgium; Heart Rhythm Management Centre, Postgraduate Program in Cardiac Electrophysiology and Pacing, Universitair Ziekenhuis Brussel-Vrije Universiteit Brussel, European Reference Networks Guard-Heart, Av. du Laerbeek 101, 1090 Jette, Brussels, Belgium; Heart Rhythm Management Centre, Postgraduate Program in Cardiac Electrophysiology and Pacing, Universitair Ziekenhuis Brussel-Vrije Universiteit Brussel, European Reference Networks Guard-Heart, Av. du Laerbeek 101, 1090 Jette, Brussels, Belgium; Heart Rhythm Management Centre, Postgraduate Program in Cardiac Electrophysiology and Pacing, Universitair Ziekenhuis Brussel-Vrije Universiteit Brussel, European Reference Networks Guard-Heart, Av. du Laerbeek 101, 1090 Jette, Brussels, Belgium; Heart Rhythm Management Centre, Postgraduate Program in Cardiac Electrophysiology and Pacing, Universitair Ziekenhuis Brussel-Vrije Universiteit Brussel, European Reference Networks Guard-Heart, Av. du Laerbeek 101, 1090 Jette, Brussels, Belgium; Heart Rhythm Management Centre, Postgraduate Program in Cardiac Electrophysiology and Pacing, Universitair Ziekenhuis Brussel-Vrije Universiteit Brussel, European Reference Networks Guard-Heart, Av. du Laerbeek 101, 1090 Jette, Brussels, Belgium; Heart Rhythm Management Centre, Postgraduate Program in Cardiac Electrophysiology and Pacing, Universitair Ziekenhuis Brussel-Vrije Universiteit Brussel, European Reference Networks Guard-Heart, Av. du Laerbeek 101, 1090 Jette, Brussels, Belgium; Heart Rhythm Management Centre, Postgraduate Program in Cardiac Electrophysiology and Pacing, Universitair Ziekenhuis Brussel-Vrije Universiteit Brussel, European Reference Networks Guard-Heart, Av. du Laerbeek 101, 1090 Jette, Brussels, Belgium; Heart Rhythm Management Centre, Postgraduate Program in Cardiac Electrophysiology and Pacing, Universitair Ziekenhuis Brussel-Vrije Universiteit Brussel, European Reference Networks Guard-Heart, Av. du Laerbeek 101, 1090 Jette, Brussels, Belgium; Heart Rhythm Management Centre, Postgraduate Program in Cardiac Electrophysiology and Pacing, Universitair Ziekenhuis Brussel-Vrije Universiteit Brussel, European Reference Networks Guard-Heart, Av. du Laerbeek 101, 1090 Jette, Brussels, Belgium; Heart Rhythm Management Centre, Postgraduate Program in Cardiac Electrophysiology and Pacing, Universitair Ziekenhuis Brussel-Vrije Universiteit Brussel, European Reference Networks Guard-Heart, Av. du Laerbeek 101, 1090 Jette, Brussels, Belgium; Heart Rhythm Management Centre, Postgraduate Program in Cardiac Electrophysiology and Pacing, Universitair Ziekenhuis Brussel-Vrije Universiteit Brussel, European Reference Networks Guard-Heart, Av. du Laerbeek 101, 1090 Jette, Brussels, Belgium; Heart Rhythm Management Centre, Postgraduate Program in Cardiac Electrophysiology and Pacing, Universitair Ziekenhuis Brussel-Vrije Universiteit Brussel, European Reference Networks Guard-Heart, Av. du Laerbeek 101, 1090 Jette, Brussels, Belgium; Texas Cardiac Arrhythmia Institute, St. David's Medical Center, 3000 N Interstate Hwy 35, Austin, TX 78705, USA; Heart Rhythm Management Centre, Postgraduate Program in Cardiac Electrophysiology and Pacing, Universitair Ziekenhuis Brussel-Vrije Universiteit Brussel, European Reference Networks Guard-Heart, Av. du Laerbeek 101, 1090 Jette, Brussels, Belgium; Heart Rhythm Management Centre, Postgraduate Program in Cardiac Electrophysiology and Pacing, Universitair Ziekenhuis Brussel-Vrije Universiteit Brussel, European Reference Networks Guard-Heart, Av. du Laerbeek 101, 1090 Jette, Brussels, Belgium; Heart Rhythm Management Centre, Postgraduate Program in Cardiac Electrophysiology and Pacing, Universitair Ziekenhuis Brussel-Vrije Universiteit Brussel, European Reference Networks Guard-Heart, Av. du Laerbeek 101, 1090 Jette, Brussels, Belgium; Heart Rhythm Management Centre, Postgraduate Program in Cardiac Electrophysiology and Pacing, Universitair Ziekenhuis Brussel-Vrije Universiteit Brussel, European Reference Networks Guard-Heart, Av. du Laerbeek 101, 1090 Jette, Brussels, Belgium; Heart Rhythm Management Centre, Postgraduate Program in Cardiac Electrophysiology and Pacing, Universitair Ziekenhuis Brussel-Vrije Universiteit Brussel, European Reference Networks Guard-Heart, Av. du Laerbeek 101, 1090 Jette, Brussels, Belgium; Heart Rhythm Department, Clinique Pasteur, 45 Av. de Lombez BP 27617 - 31076, 31300 Toulouse, France; Cardiac Surgery Department, Universitair Ziekenhuis Brussel-Vrije Universiteit Brussel, Av. du Laerbeek 101, 1090 Jette, Brussels, Belgium; Texas Cardiac Arrhythmia Institute, St. David's Medical Center, 3000 N Interstate Hwy 35, Austin, TX 78705, USA; Heart Rhythm Management Centre, Postgraduate Program in Cardiac Electrophysiology and Pacing, Universitair Ziekenhuis Brussel-Vrije Universiteit Brussel, European Reference Networks Guard-Heart, Av. du Laerbeek 101, 1090 Jette, Brussels, Belgium; Heart Rhythm Management Centre, Postgraduate Program in Cardiac Electrophysiology and Pacing, Universitair Ziekenhuis Brussel-Vrije Universiteit Brussel, European Reference Networks Guard-Heart, Av. du Laerbeek 101, 1090 Jette, Brussels, Belgium; Heart Rhythm Management Centre, Postgraduate Program in Cardiac Electrophysiology and Pacing, Universitair Ziekenhuis Brussel-Vrije Universiteit Brussel, European Reference Networks Guard-Heart, Av. du Laerbeek 101, 1090 Jette, Brussels, Belgium

**Keywords:** Atrial fibrillation, Pulsed field ablation, Substrate, Left atrial appendage, Atrial flutter, Mapping, electrograms

## Abstract

**Aims:**

Rhythm control of non-paroxysmal atrial fibrillation (AF) is significantly more challenging, as a result of arrhythmia perpetuation promoting atrial substrate changes and AF maintenance. We describe a tailored ablation strategy targeting multiple left atrial (LA) sites via a pentaspline pulsed field ablation (PFA) catheter in persistent AF sustained beyond 6 months (PerAF > 6 m) and long-standing persistent AF (LSPAF).

**Methods and results:**

The ablation protocol included the following stages: pulmonary vein antral and posterior wall isolation plus anterior roof line ablation (Stage 1); electrogram-guided substrate ablation (Stage 2); atrial tachyarrhythmia regionalization and ablation (Stage 3). Seventy-two [age:68 ± 10years, 61.1%males; AF history: 25 (18–45) months] patients with PerAF > 6 m (52.8%) and LSPAF (47.2%) underwent their first PFA via the Farapulse^TM^ system. LA substrate ablation (Stage 1 and 2) led to AF termination in 95.8% of patients. AF organized into a left-sided atrial flutter (AFlu) in 46 (74.2%) patients. The PFA catheter was used to identify LA sites showing diastolic, low-voltage electrograms and entrainment from its splines was performed to confirm the pacing site was inside the AFlu circuit. Left AFlu termination was achieved in all cases via PFA delivery. Total procedural and LA dwell times were 112 ± 25 min and 59 ± 22 min, respectively. Major complications occurred in 2 (2.8%) patients. Single-procedure success rate was 74.6% after 14.9 ± 2.7 months of follow-up; AF-free survival was 89.2%.

**Conclusion:**

In our cohort, PFA-based AF substrate ablation led to AF termination in 95.8% of cases. Very favourable clinical outcomes were observed during >1 year of follow-up.

## Graphical Abstract

**Graphical Abstract:**
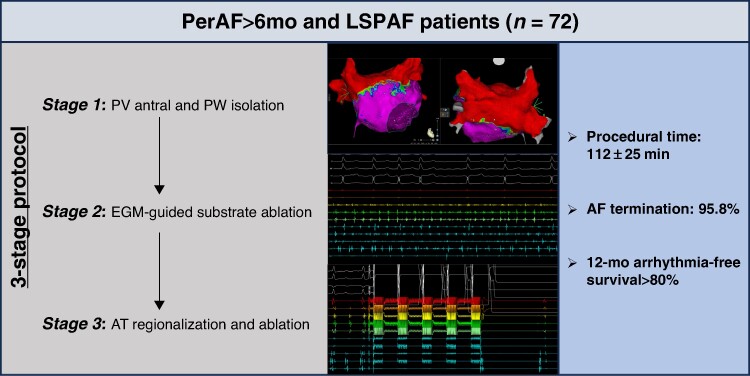

**What’s new?**

- An ablation strategy targeting PVs, PW and the LA substrate involved in AF maintenance was safely achieved via the multispline Farawave^TM^ catheter and successfully contributed to AF termination in 95.8% of patients.
- The ablation protocol was performed in all patients in a relatively short time.
- Arrhythmia-free and AF-free survival rates at 12 months were ∼80 and 90%, respectively.

## Introduction

Atrial arrhythmia perpetuation promotes electrical and structural remodelling predisposing the atria to sustain atrial fibrillation (AF). Therefore, rhythm control of non-paroxysmal AF is significantly more challenging; furthermore, sources of ectopic beats outside the pulmonary veins (PVs) are more likely to precipitate AF initiation, making the PVs a suboptimal ablation target.^1–5^

In this perspective, an effective strategy should aim at targeting the main sources of triggers involved in arrhythmia initiation, as well as modifying the diseased atrial substrate contributing to AF maintenance.^6^

Several ablation approaches have been described but none of them showed a clear superiority in randomized studies.^7–12^ A possible explanation lies in the intrinsic limitations of radiofrequency (RF) energy in creating durable lesions without increasing the risk of collateral thermal injury to adjacent structures (e.g. oesophagus, nerves).^13–15^ Additionally, severe left atrial (LA) scarring due to extensive RF ablation may lead to reduced LA compliance (stiff LA syndrome) and manifest with symptoms of pulmonary hypertension.^16^

Pulsed field ablation (PFA) has emerged as a novel, non-thermal energy source for cardiac ablation. Preclinical and clinical studies have shown a remarkable lesion durability and safety profile,^5,16–18^ with no known risk of collateral damage to surrounding structures.

Among the PFA devices with regulatory approval, the multielectrode Farawave^TM^ catheter (Farapulse^TM^-Boston Scientific Inc., Marlborough, Massachusetts, USA) has been originally designed for PVI but also used for ablation of extra-pulmonary site.^19–21^

Herein we describe the feasibility and electrophysiological findings of a tailored ablation strategy targeting multiple LA sites via the multielectrode Farawave catheter in patients with persistent AF sustained beyond 6 months (PerAF > 6 m) and long-standing persistent AF (LSPAF). We developed a 3-stage workflow to standardize and guide the procedure.

## Methods

### Study population

We enrolled consecutive patients referred for first-time ablation of PerAF > 6 m and LSPAF. LSPAF was defined according to the 2020 ESC Guidelines for the diagnosis and management of AF,^22^ as continuous AF of >12 months in duration.

Procedures were performed via the Farapulse^TM^ PFA system (Boston Scientific, Marlborough, Massachusetts, USA) by a total of 7 operators.

All patients signed a written informed consent for the ablation procedure and participation to a local registry for research purposes. The Institutional Review Board reviewed and approved the registry for prospective data collection and observational studies.

### Ablation strategy

All procedures were performed under general anaesthesia and uninterrupted oral anticoagulation.

Two right groin accesses under ultrasound guidance were used to achieve transseptal access via an 8.5F SL0 fixed sheath (Abbott, St Paul, MN, USA) and advance a 10-pole catheter into the right atrium (RA) or the coronary sinus (CS).

Heparin was administered at the time of transseptal access and during the procedure, when needed, to maintain an activated clotting time between 300 and 350 s.

The SL0 sheath was exchanged over-the-wire with the Faradrive^TM^ sheath and a 31 mm (distal diameter) PFA catheter Farawave^TM^ was advanced into the LA. The PFA catheter has 5 splines and 20 electrodes (4 per spline). Sensing and pacing occur via the equatorial electrodes, resulting in 5 bipoles with an inter-electrode distance of 16 and 17 mm when the catheter is deployed in flower and basket configuration, respectively. Only 31 mm (distal diameter) catheters were used for the purpose of this study.

PFA was delivered at a voltage of 2.0 kV.

**Figure 1:**
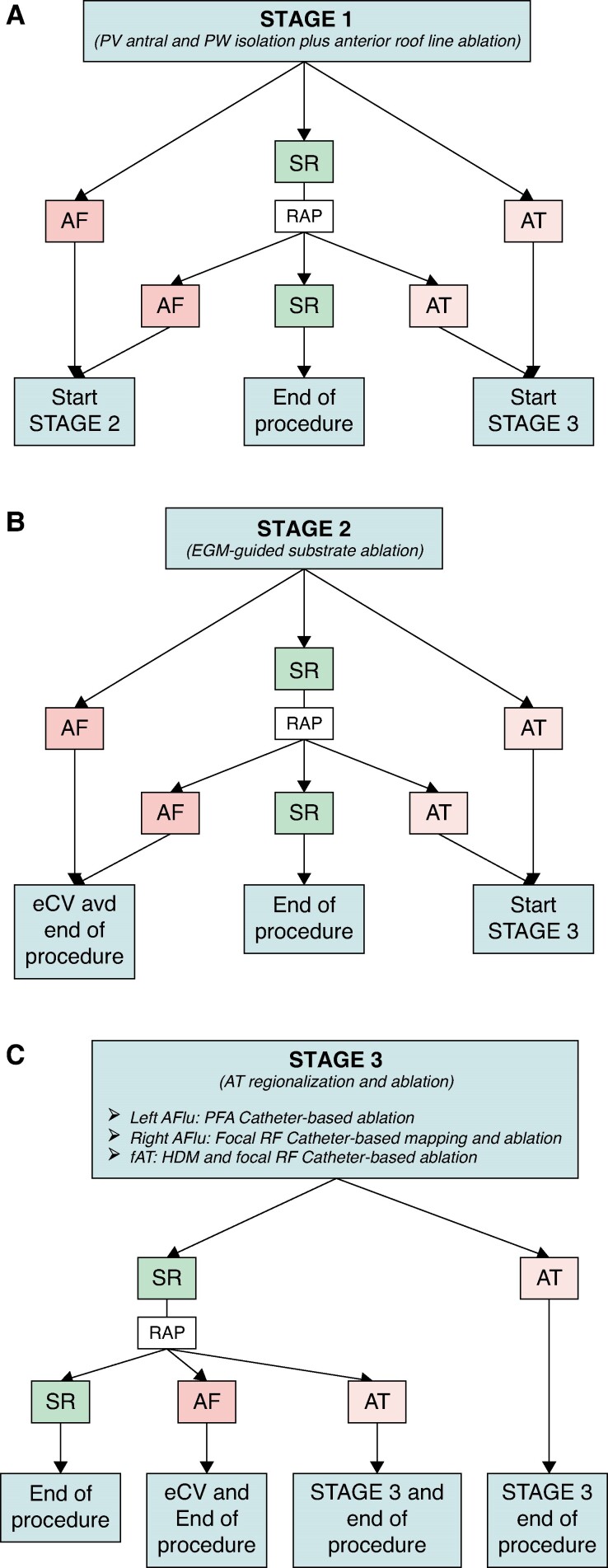
Ablation workflow. Flow chart summarizing the workflow for Stage 1 (*A*), Stage 2 (*B*), and Stage 3 (*C*). AF, atrial fibrillation; AFlu, atrial flutter; AT, atrial tachyarrhythmia; eCV, electrical cardioversion; EGM, electrogram; fAT, focal atrial tachycardia; HDM, high-density mapping; PV, pulmonary vein; PW, posterior wall; RAP, rapid atrial pacing; RF, radiofrequency; SR, sinus rhythm.

A detailed description of the procedural stages is depicted below and in *Figure 1*. No high-density mapping was required for Stages 1 and 2, and considered on a case basis for Stage 3 according to the underlying rhythm.

*-Stage 1: PV Antral and Posterior Wall (PW) Isolation plus Anterior Roof Line Ablation*: PV antral isolation was performed, as previously described,^23^ via at least four pairs of applications (two pairs in basket and two pairs in flower configuration, each pair at approximately 36° rotation from the other).

Isolation of the PW lying in between the PVs was achieved with the PFA catheter deployed into a flower pose and the wire retracted into the catheter to optimize contact. At least a pair of applications was delivered at each position with an overlap of ∼50% of the catheter surface per position.

**Figure 2:**
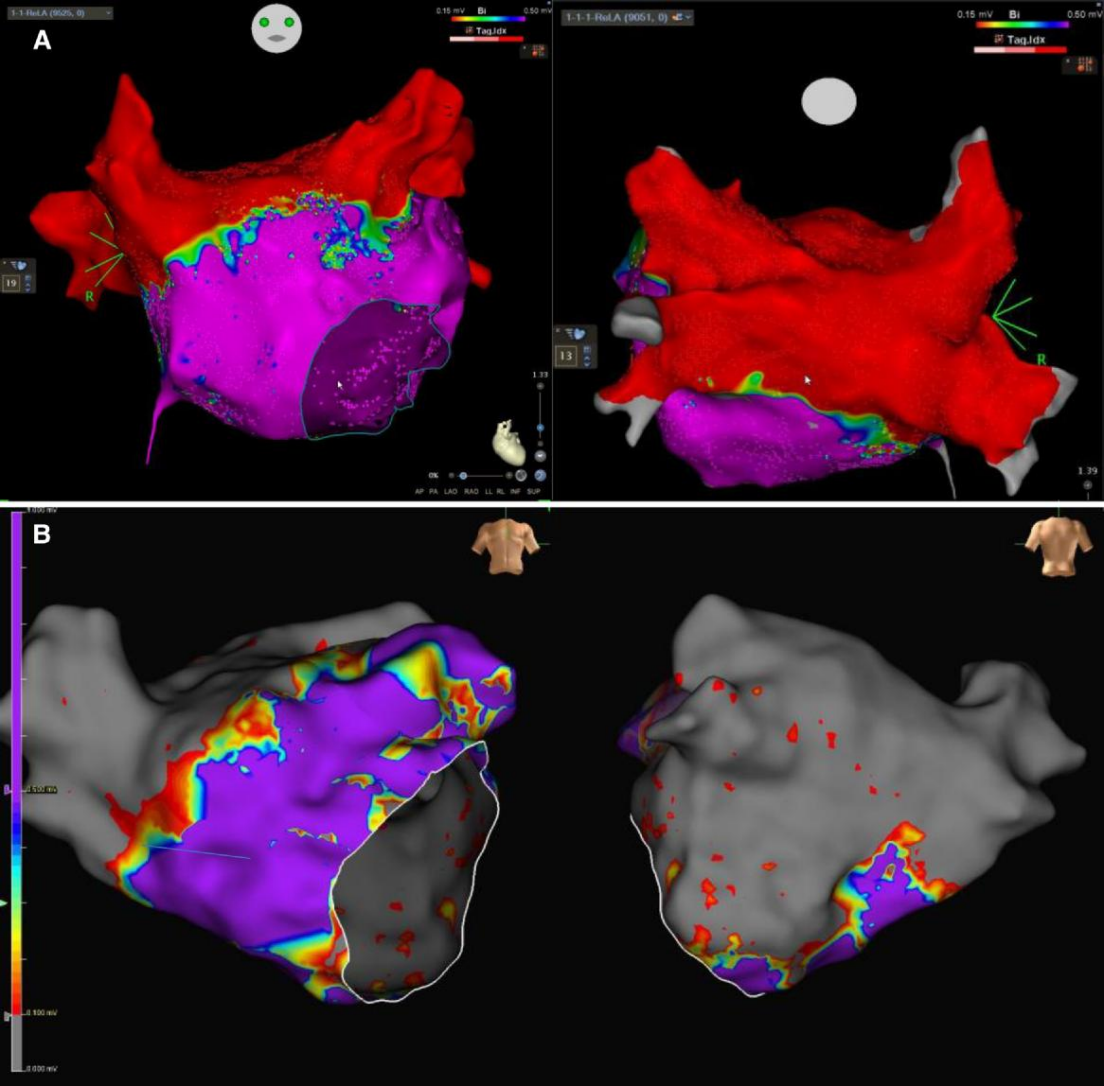
Anterior and posterior views of an electroanatomic (voltage) map performed after sinus rhythm restoration with Stage 1 (*A*, Carto^TM^ 3 V7 System, Biosense Webster, Irvine, CA, USA) and with Stage 1, 2, and 3 (*B*; EnSite^TM^ X EP System, Abbott, St Paul, MN, USA).

Anterior LA roof ablation was performed by connecting the right and left superior PVs and the PW, (*Figure 2A*) with the aim of creating an additional line of block for PW isolation.

Stage 1 ablation endpoint was complete isolation with evidence of unexcitability with high-output pacing >20 mA from the PFA catheter. Stage 1 workflow and progression to Stage 2 and 3 are summarized in *Figure 1*.

**Figure 3:**
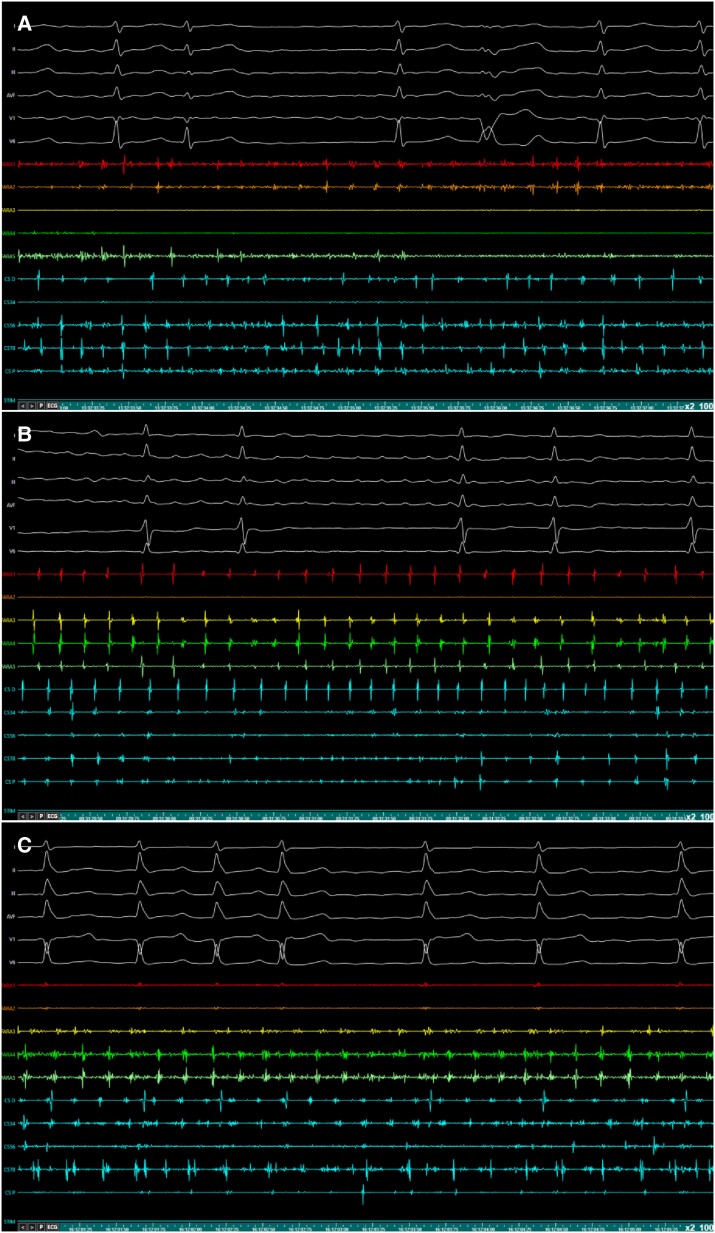
Examples of EGMs of interest for AF substrate ablation. Recordings from the Farawave^TM^ bipoles (Fara 1–5) show (*A*) continuous, low voltage (<0.25 mV) electrical activity with no isoelectric line on FARA 1–2,5 bipolar recordings, (*B*) rapid, non-fractionated EGMs on FARA 1,3–5 recordings, and (*C*) fast repetitive bursts of fractionated EGMs on FARA 3–5 bipolar recordings. Of note, some bipoles of the PFA catheter do not display any LA recordings since the device is anchored to an existing non-conduction boundary before ablation. CS recordings are reported in light blue. CL, cycle length; CS, coronary sinus; EGM, electrogram; FARA, farawave^TM^ catheter; PFA, pulsed-field ablation.

*-Stage 2: Electrogram (EGM)-guided Substrate Ablation (Fig. 2B and 3):* Bipolar recordings from the PFA catheter deployed into flower configuration were filtered at 40–400 Hz. EGMs of interest (*Figure 3*) had the following morphologies:^24–26^ (i) continuous, low voltage (<0.25 mV) electrical activity with no isoelectric line; (ii) bursts of fractionated EGMs; (iii) rapid, non-fractionated EGMs with a CL < 120 ms.

A sequential approach was adopted to systematically map and target LA anatomical sites in the following order^27^: (i) the IAS anterior to the right PVs and the inferior LA, such as the area encompassed by the IAS (right), the PW (superior), the mitral annulus (inferior), and the postero-lateral ridge (lateral); (ii) the endocardial aspect of the CS/peri-mitral area; (iii) the postero-lateral ridge from the mitral edge to its superior aspect facing the LAA; (iv) the ostium and the base of the LAA; (v) the anterior wall. Further details on the substrate ablation strategy are reported in the Supplementary material online, materials. Stage 2 workflow and progression to Stage 3 are summarized in *Figure 1*.

*-Stage 3: Atrial Tachyarrhythmia (AT) Regionalization and Ablation:* AT was defined as an organized atrial rhythm with a stable bi-atrial activation pattern. This term included either focal AT (fAT) and macroreentrant arrhythmias [atrial flutters (AFlu)]. An AFlu was suspected if the AT cycle length was stable and entrainment through the decapolar and/or the PFA catheter from at least 2 segments showed post-pacing intervals not exceeding the tachycardia cycle length by 20 ms. AFlu regionalization was achieved with entrainment through the decapolar catheter placed sequentially into the high lateral right atrium or the CS aiming at discriminating between right- and left-sided reentrant ATs.^28^

**Figure 4:**
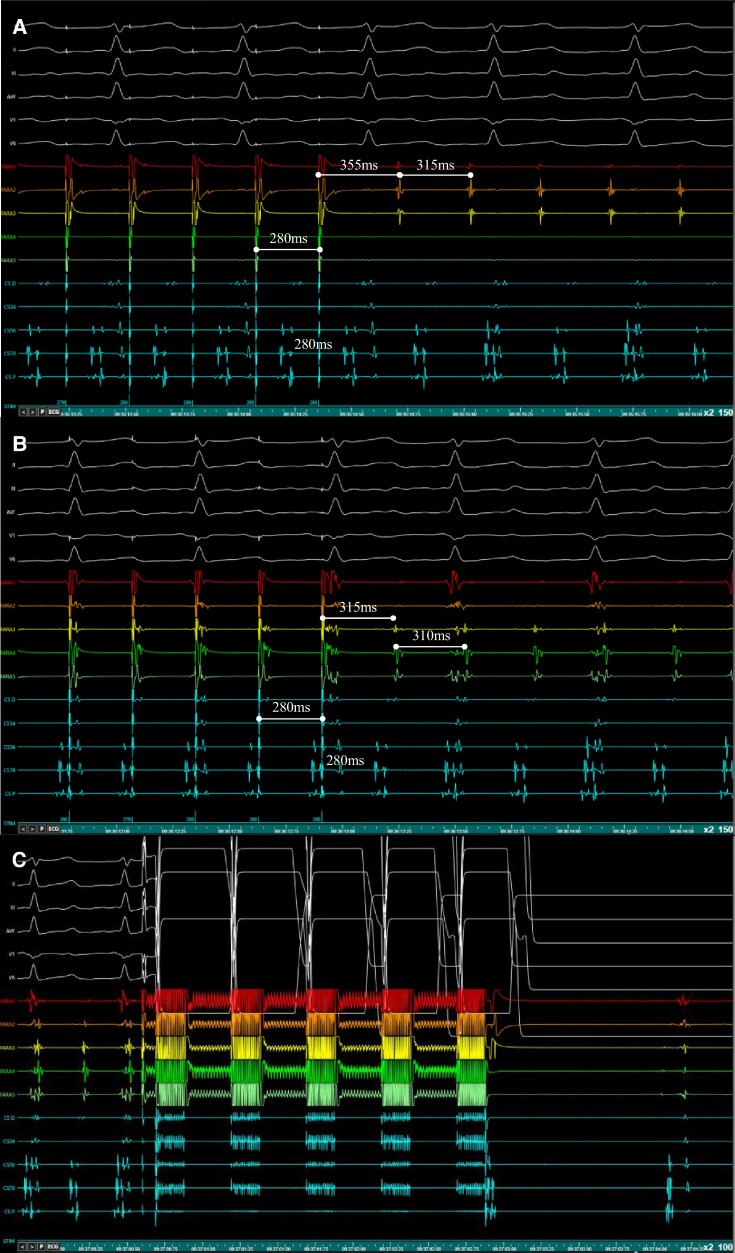
Entrainment from the PFA Catheter. Baseline AFlu CL: 315 ms (*A*) Entrainment from FARA2 suggested that the electrodes are not within the circuit (PPI-TCL = 40 ms). (*B*) The Farawave catheter was repositioned. Entrainment from FARA3 confirmed that the site is within the reentrant circuit (PPI-TCL: 5 ms). (*C*) A single PFA application at this site led to immediate AFlu termination. CS recordings are reported in light blue. AFlu, atrial flutter; CL, cycle length; EGM, electrogram; PFA, pulsed field ablation; PPI, post-pacing interval; TCL, tachycardia cycle length.

In the case of a left-sided AFlu, the PFA catheter was used to identify slow conduction sites showing diastolic low-voltage, fractionated electrograms on one or more splines. If entrainment confirmed participation of the site to the reentrant circuit, PFA was delivered (*Figure 4*).

If PFA led to AFlu termination, attention was paid to anchor the site of ablation to one or more non-conducting boundary aiming at avoiding the development of iatrogenic slow-conducting sites.

In case of a right-sided AFlu, a point-by-point ablation catheter was used to map and ablate the reentrant circuit.

If a fAT was suspected, a high-density mapping catheter was used to localize the arrhythmia and ablation at the site of origin was performed in a point-by-point fashion.

Stage 3 workflow is summarized in *Figure 1*.

- *Technical Considerations:*

(i) RAP was performed at least 3 times from the proximal dipoles of the decapolar catheter placed into the CS at a cycle length down to 180 ms or to the refractory period. (ii) Nitrates were administered before delivering PFA to sites adjacent to a coronary artery (e.g. peri-mitral area). (iii) If electrograms of interest with optimal entrainment were identified but bystander high voltage electrograms were present on the other dipoles of the PFA catheter, the catheter was repositioned around the area of interest to avoid concomitant ablation of adjacent healthy tissue.

### Follow-up, study endpoints and definitions

Details of the post-procedural and follow-up management are reported in the Supplementary material online, methods. Total procedure time was calculated from femoral puncture to catheter removal. LA dwell time was considered as the time the PFA catheter was kept in the LA.

Primary safety endpoint included any major procedure- and technology-related complications occurring within 7 days post-ablation.

Primary efficacy endpoint was defined as freedom from any AT (AF, Aflu and fAT) lasting >30 s, irrespective of symptoms, occurring after a 4-week blanking period.

### Statistical analysis

Categorical and continuous data were reported as absolute values (percentage) and mean ± standard deviation or median and interquartile range (IQR) for non-normal data. Kaplan-Meier curves were generated for arrhythmia-free survival. All tests were two-sided, and a *P*-value <0.05 was considered statistically significant. Analyses were performed with IBM SPSS Statistics 25.0 (IBM SPSS Inc, Chicago, IL, USA) and STATA 18.0 (StataCorp, College Station, TX, USA).

## Results

Out of 72 patients with a diagnosis of PerAF > 6 m or LSPAF underwent their first PFA via the multielectrode Farapulse^TM^ system (*Table 1*, *Figures 1* and *4*); the mean age was 68 ± 10years and males accounted for 61.1%. Out of 38 (52.8%) patients had PerAF > 6 m and 34 (47.2%) LSPAF. Median AF history was 25 months (IQR: 18–45) and median duration of current/latest AF episode was 12 months (IQR: 7–15).

**Table 1:** Baseline characteristics

Demographics	Overall (*n* = 72)
Age, y	68 ± 10
Male gender	44 (61.1)
***Risk factors***	
Hypertension	56 (77.8)
Diabetes mellitus	17 (23.6)
Obstructive sleep apnea	16 (22.2)
BMI, km/m^2^	29 ± 5
Vascular disease	20 (27.8)
CHF	23 (31.9)
History of stroke/TIA	7 (9.7)
CHA2DS2-VASc	3 [2–4]
HAS-BLED	2 [2–3]
***Transthoracic echocardiography***	
LA diameter, mm	49 ± 8
LVEF, % [range]	51 ± 9 [25–60]
LVEF ≤35%	8 (11.1)
***AF characteristics***	
AF history, m	25 [18–45]
Duration of current/latest episode	12 [7–15]
LSPAF	34 (47.2)
Previous cardioversions	66 (91.7)
***AADs***	
Class I	24 (33.3)
Class II	57 (79.2)
Class III	32 (44.4)

Values are expressed as mean ± standard deviation, median [interquartile range], or *n* (%).

AAD, anti-arrhythmic drug; AF, atrial fibrillation; BMI, body mass index; CHF, chronic heart failure; y; years; LA, left atrial; LSPAF, long-standing persistent atrial fibrillation; LVEF, left ventricular ejection fraction; PVI, pulmonary vein isolation.

*Stage 1: PV antral and PW isolation plus Anterior Roof Line Ablation:* After successful PVI, PW isolation and roof line ablation were achieved after a median of 19 (IQR: 14–23) applications in flower pose.

Seven patients had AF termination during PW (*n* = 6) or roof line (*n* = 1) ablation; sinus rhythm (SR) restoration occurred in 4 patients, whereas AF organized into an AT in the other 3 (*Figure 4*). RAP led to AT induction in 1 of 4 patients in SR (*Table 2*).

**Table 2:** Procedural details

Characteristics	Overall (*n* = 72)
Procedural time, min	112 ± 25
Fluoroscopy time, min	37 ± 15
LA dwelling time, min	59 ± 22
Total number of PFA applications	84 ± 12
**Stage 1 (*n* = 72)**	
AF termination with ablation	7 (9.7)
*SR restoration*	*4/7*
*Re-inducibility*	*1/4*
Site of AF termination	
*PW*	*6* (*85.7)*
*Roof 1 (14.3)*	
**Stage 2 (*n* = 65)**	
Sites of substrate ablation	
*IAS*	*60* (*92.3)*
*PL ridge (mitral edge)*	*59* (*90.8)*
*Inferior LA/Mid. CS*	*56* (*86.1)*
*Inferior LA/Prox.CS*	*41* (*63.1)*
*PL ridge (PV Edge)*	*31* (*47.7)*
*Anterior wall*	*11* (*16.9)*
*LAA*	*5* (*7.7)*
AF termination with ablation	62 (95.4)
*SR restoration*	*6* (*9.7)*
**Stage 3 (*n* = 62)**	
AT at beginning of stage 3	
*Right-sided AFlu*	*16* (*25.8)*
*Left-sided AFlu*	*46* (*74.2)*
Overall ATs mapped and ablated	71
Pts requiring a focal Abl. catheter	25 (40.3)
Pts requiring HDM	8 (12.9)
SR restoration with ablation	62 (100)
Final re-inducibility	0 (0.0)

Values are expressed as mean ± standard deviation, median [interquartile range], or *n* (%).

Abl, ablation; AF, atrial fibrillation; AFlu, atrial flutter; CS, coronary sinus; EGM, electrogram; HDM, high-density mapping; IAS, inter-atrial septum; LA, left atrial; LAA, left atrial appendage; PFA, pulsed field ablation; PL, postero-lateral; Pts, patients; PW, posterior wall; SR, sinus rhythm.

*Stage 2: EGM-guided Substrate Ablation:* Stage 2 was performed in 65 patients who remained in AF after Stage 1. All patients had at least 3 or more LA sites of interest; their distribution is summarized in *Table 2*.

Stage 2 led to AF termination in 62 (95.4%) of 65 patients (*Table 2*); of them, SR restoration occurred in 6 (9.7%) patients, whereas AF organized into an AT in 56 (90.3%) others. AF termination occurred while ablating the mitral edge of the postero-lateral ridge (*n* = 25; 40.3%; *Figure 2*), the inferior left atrium above the proximal CS (17; 27.4%), the IAS (*n* = 14; 22.6%), the superior aspect of the postero-lateral ridge (*n* = 4; 6.5%), the base of the appendage (*n* = 2; 3.2%).

Three (4.6%) of 65 patients who remained in AF had SR restored via electrical cardioversion.

*Stage 3: AT Regionalization and Ablation:* Stage 3 was started in 62 patients (58 from Stage 2, including 2 of 6 in SR who had an AT induced with RAP, plus 4 from Stage 1).

Pacing maneuvers sequentially performed from multiple RA and LA sites suggested a right or left origin of the AFlu in 16 (25.8%) and 46 (74.2%) patients, respectively (*Table 2*).

A point-by-point ablation catheter was used to map and ablate right AFlus [all cavotricuspid isthmus dependent] with restoration of SR in all 16 cases.

For left-sided flutters (*n* = 46), SR restoration was achieved in 39 (84.8%) patients by means of PFA at sites detected via entrainment from the pentaspline catheter.

The sites of termination were the mitral edge of the postero-lateral ridge (*n* = 19; 48.7%), the anterior roof next to the right superior PV (*n* = 9; 23.1%), the inter-atrial septum (*n* = 7; 17.9%), the base of the appendage (*n* = 4; 10.3%). According to the entrainment results and sites of termination, the expected AFlu location involved the mitral annulus (*n* = 23), the anterior/anteroseptal wall (*n* = 13), both atria (*n* = 3).

In the remaining 7 patients, the AT organized into a different arrhythmia after PFA. RAP led to AFlu/AT re-initiation in 5 patients. Overall, 67 AFlus and 4 focal ATs were detected and ablated. A focal ablation catheter was required in 25 (34.7) of 72 patients, including 8 (11.1) undergoing additional high-density mapping. Electroanatomical mapping identified 2 focal ATs from the LAA, 1 from the anterior wall, 1 from the septum, as well as the circuits of 1 peri-mitral, 2 anterior wall dependent, and 1 bi-atrial flutters.

### Procedural data and primary safety endpoint

Total procedural and LA dwelling times were 112 ± 25 min and 59 ± 22 min, respectively (*Table 2*). Fluoroscopy time was 37 ± 15 min. The primary safety endpoint occurred in 2 (2.8%) patients with pre-existing left ventricular systolic dysfunction (EF < 35%). These patients required inotropic therapy due to acute heart failure with low cardiac output and hypotension (systolic blood pressure <90 mmHg). Hemodynamics normalized within 24 h and both patients were discharged after 2 and 3 additional hospital days.

No instances of clinically relevant acute kidney injury were observed.

Transient ST-segment elevation was documented in four patients after peri-mitral PFA; timely nitrate administration led to ECG normalization within 10 min. Of note, all ST-segment elevation episodes occurred in the first 20 patients of the series, when administration of prophylactic nitrates before PFA to sites adjacent to a coronary artery had not been adopted yet.

One patient developed a groin hematoma that was treated conservatively.

### Primary efficacy endpoint and echocardiographic assessments

**Figure 5:**
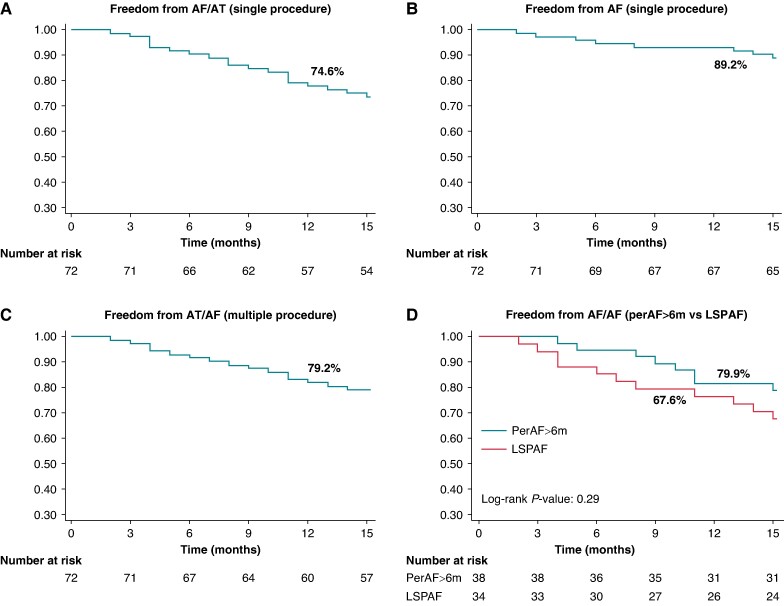
Kaplan-Meier analysis. Kaplan-Meier analysis showing freedom from AF/AT (*A*), and AF (*B*). (*C*) and (*D*) depict arrhythmia-free survival after multiple procedures and in PerAF > 6 m vs. LSPAF patients (log-rank *P*-value: 0.29), respectively. AF, atrial fibrillation; AT, atrial tachyarrhythmia; FU, follow-up; LSPAF, long-standing persistent AF; PerAF > 6m, persistent AF sustained beyond 6 months in duration.

Echocardiographic data on LAA mechanical activity and LA function are reported in Supplementary material online, results and  *Figure S1*. All patients completed one year of follow-up (mean follow-up: 14.9 ± 2.7 months). Three (4.2%) patients had an arrhythmic recurrence during the one-month blanking period and were kept on AAD therapy during follow-up. Overall, 53 (74.6%) patients remained arrhythmia-free after a single procedure (*Figure 5A*), with no difference between persistent and LSP AF subpopulations (79.9% vs. 67.6%; log-rank *P*-value: 0.29; *Figure 5D*). Among the 13 (18.2%) patients with recurrence, AF relapse occurred in 8 (freedom from AF: 89.2%, *Figure 5B*). Four patients (*Figure 5C*) underwent redo ablation showing durable PV isolation in all patients. Two patients had a roof-dependent AFlu at presentation, with evidence of reconnection of the PW and roof. Two others had a peri-mitral flutter. No thromboembolic events were documented.

## Discussion

Herein we report the feasibility of a novel ablation strategy for PerAF > 6 m and LSPAF via a commercially available multielectrode PFA catheter. We also describe a workflow with prespecified endpoints aiming at a structured ablation approach. Our main findings are the following:

1. An ablation strategy targeting PVs, PW and the LA substrate involved in AF maintenance was safely achieved via the multispline Farawave^TM^ catheter. AF termination/organization was successfully achieved in 95.8% of patients;
2. We described a strategy for regionalization of organized ATs with a stable bi-atrial activation pattern. Regionalization was based on multiple entrainment maneuvers through a decapolar catheter alternatively placed into the RA and CS, as well as from the multielectrode PFA catheter in the LA. PFA targeting sites of interest in the LA detected and confirmed with entrainment via the Farawave^TM^ catheter led to arrhythmia termination in all cases;
3. The ablation protocol was performed in all patients in a relatively short time (total procedural time: 112 ± 25 min);
4. Arrhythmia-free and AF-free survival rates at 12 months were ∼80 and 90%, respectively.

PFA has recently emerged as a safe and effective non-thermal energy source for AF ablation. Its main advantage lies in a remarkable safety profile. Specifically, a very low to no risk of collateral tissue damage (e.g. nerves, arteries and oesophagus), as well as a more efficient and durable lesion formation were confirmed in trials and large registries.^5,17,19,29^

The Farawave^TM^ has been the first PFA catheter to receive regulatory (CE-mark) approval and has been specifically designed to target the PVs. Its flower configuration also perfectly adapts to ablating the LAPW, as demonstrated in a recent phase 3 study showing 100% durable PW isolation at invasive remapping after a median of 82 days post-ablation.^19^ Nonetheless, our knowledge on the use of this catheter for extra-pulmonary ablation is still anecdotal and limited to case reports or modest observational studies.^3,17,21,30^

Herein we describe a novel workflow to target the LA substrate as an adjunctive ablation target of the Farawave^TM^ catheter in PerAF > 6 m and LSPAF. Our findings suggest that EGM-guided substrate modification can be effectively achieved with the Farawave^TM^ catheter. As a matter of fact, we observed a 95.8% rate of AF termination/organization and a freedom of ∼90%. From AF at 12 months of follow-up.

We believe there are at least three main advantages in adopting a PFA strategy to target the substrate that maintains AF.

First, PFA lesion formation is faster, safer and more durable than RF ablation. Previous studies on substrate modification reported clinical outcomes similar to what we observed in our series. As an example, the Marshall-PLAN reported a 79% arrhythmia freedom after a single procedure in a population of PersAF and LSPAF (24%) patients.^31^ Di Biase et al. previously observed a success rate at 1 year of 56% in LSPAF-only patients undergoing non-PV trigger ablation and empirical LA appendage isolation.^32^ Similar findings were described by Tilz et al. among LSPAF undergoing additional complex fractionated atrial electrogram and LA linear ablation.^33^ In the above-mentioned experiences, very long procedural times were reported (range: 180–280 min),^2,25,34,35^ as compared with 112 ± 25 min observed in our series. More efficient procedures mean lower risk of periprocedural complications, which is a key factor in PerAF and LSPAF patients who notoriously have a high burden of cardiovascular comorbidities, and have also a positive impact on healthcare expenditure.^36^ PFA has been recently demonstrated to contribute to more predictable procedural times, as well as better outcomes of PV isolation-only in PerAF patients, when compared with RF or cryoablation.^5,37^

Second, extensive thermal ablation has been demonstrated to contribute to an impaired LA reservoir function. Impaired LA function results from post-necrosis reparative fibrosis, which may increase the risk of stiff LA syndrome.^38^ PFA lesions do not alter the extracellular matrix, which represents a critical factor for LA diastolic function recovery in the chronic stage.^16,39^ Of note, a significant LA mechanical contraction improvement was noted in a subgroup of our cohort after ∼6 months of follow-up. These findings were present even among patients with more extensive ablations or when a significantly impaired mechanical function was documented at baseline.

Third, achieving more durable and homogeneous lesions is of utmost importance to improve outcomes and prevent the onset of ATs of iatrogenic etiology. RF-based extra-PV ablation is characterized by high rates of conduction recovery, which may contribute to suboptimal rhythm control.^13,40^ As an example, PW reconnection has been reported to occur in up to 80% of patients undergoing post-RF ablation repeat procedures.^40^ Data on extra-PV lesion durability after PFA are promising but still limited, with a preliminary phase-3 study showing 100% durable PW isolation at chronic remapping.^19^

The main drawback of using the Farawave^TM^ catheter for substrate modification, as well as for left AFlu mapping and ablation, is its dimensions. The device is available in two different sizes (31 and 35 mm), which have been chosen to optimize PV antral isolation and fit a wide variety of venous anatomies. However, being a single-shot device, it is not the optimal choice when ablation outside the PVs/PW is needed. In this perspective, we adopted a few measures to overcome these limitations. Only 31 mm devices were used in order to reduce the risk of collateral myocardial tissue ablation and improve device-tissue interface and contact. For AF substrate ablation, a sequential regional pattern to systematically map and ablate EMGs of interest was adopted aiming at sparing/limiting ablation of the LAA due to its role in booster pump function. Also, substrate mapping was performed with the PFA catheter systematically anchored to an existing non-conduction boundary (e.g. PW, PVs, mitral annulus) and progressively moved away from it (*Figure 3*). The rationale behind this approach is primarily to prevent development of slow-conducting sites, which may be associated with iatrogenic reentrant arrhythmias.

Similarly, if a left-sided AFlu was observed, entrainment from the bipole of interest was used to confirm participation of the site to the reentrant circuit. However, if high voltage electrograms were also present on one or more other bipoles of the PFA catheter, the placement of the ablation catheter was optimized by repositioning the device around the area of interest before PFA was delivered (*Figure 4*). This strategy was developed aiming at preventing inadvertent ablation of bystander healthy myocardial tissue. Some of these limitations would be overcome if future device developments allowed the operator to select specific splines, thereby activating or deactivating them for a targeted PFA delivery. Additionally, the option of initial application of low-energy reversible pulse on selected splines would also allow testing of the importance of the target site in the maintenance of the arrhythmia.

Last, recent reports have described PFA-induced hemolysis potentially contributing to acute kidney injury. This phenomenon appears to be more likely with an increasing PFA application number and lack of catheter-tissue contact.^41,42^ None of our patients developed any clinically relevant acute kidney injury irrespective of the high mean number of PFA applications. This result was likely due to a fluid infusion protocol after ablation, as we described in a recent manuscript.^42^

## Limitations

Our study has several limitations that need to be acknowledged. First, this is a single-center, single-arm, non-randomized study with all the inherent limitations and biases associated with this design. Second, EGM definition and mapping were adapted from previous studies to a novel device that has not been originally designed for this purpose. Furthermore, as sensing occurs via bipoles with an inter-electrode distance of 16/17 mm, the sensitivity and specificity of substrate mapping and arrhythmia regionalization with the pentaspline catheter are not comparable to previous studies with high-density mapping systems and need further validation. Third, follow-up echocardiographic data on LA function are available in less than half of our patients; similarly, no transoesophageal echocardiography was performed during follow-up to assess LAA function.

Fourth, large-area PFA application may potentially lead to arrhythmia termination due to local capture/cardioversion effect. However, flutter reinduction and recurrence rates was low during follow-up suggesting that the target sites played an important arrhythmogenic role. Fifth, scar and low-voltage substrate zones were mapped in only a limited number of patients. Future integration of the pentaspline catheter into electroanatomical mapping systems may overcome this issue. Last, our workflow was not tested against other ablation strategies with/without PFA (e.g. PVI alone or in combination with other targets). Also, only one device (Farawave^TM^ 31 mm) was used in our series. Therefore, these findings cannot be generalized to other technologies and no conclusions can be drawn on the observed efficacy compared with other ablation workflows.

## Conclusions

PFA-based AF substrate ablation led to AF termination in 95.8% of cases. The multispline catheter was successfully used to localize, entrain, and ablate left-sided AFlus resulting from AF termination, without the need for an additional focal RF catheter. Favorable clinical outcomes were observed at one year. Future randomized trials assessing the safety and efficacy of this strategy in a larger population are warranted.

## Supplementary Material

euae246_Supplementary_Data

## Data Availability

The data underlying this article will be shared upon reasonable request by the corresponding author.
