# Supplementary material for: Multielectrode catheter-based pulsed field ablation of persistent and long-standing persistent atrial fibrillation
Source: Europace. 2024 Oct 1;26(10):euae246. doi: 10.1093/europace/euae246 (PMC11498055; doi:10.1093/europace/euae246)

Multielectrode Catheter-Based Pulsed-Field Ablation of Persistent and Long-Standing Persistent Atrial Fibrillation

Della Rocca DG, Chierchia GB, et al.

Supplemental Methods.

Ablation System

Pre- and Peri-Procedural Management

EGM -guided Substrate Ablation

Hospital Management and Follow-up.

Supplemental Results.

Echocardiographic Assessments

Supplemental References.

Supplemental Figure.

Supplemental Figure 1

Supplemental Methods.

Ablation System.

The ablation system has been extensively described in previous publications, and consists of 1) the Farastar™ pulse generator, which delivers a proprietary biphasic, bipolar waveform with an output voltage of up to 2.0kV, 2) the 13F steerable sheath Faradrive™, and 3) the PFA catheter Farawave™, a 115cm over-the-wire catheter with 20 electrodes distributed over five splines which can be deployed into a basket or a flower configuration.

EGM-guided Substrate Ablation.

The PFA catheter deployed into a flower configuration was used to detect EGMs displaying spatio-temporal dispersion on multiple bipoles at discrete anatomical sites ["dispersion zones" (DZs)]. DZs were defined, according to previous studies [1,2], as sites where a cluster of EGMs spreads over the whole AF cycle length in at least 3 bipoles of the PFA catheter (Figure 3). The aim of EGM ablation was achieving abolition of local electrograms and conversion of AF into an organized AT with a defined cycle length.

Substrate mapping and ablation with the multielectrode PFA catheter aimed at anchoring 2 splines to an existing non-conduction boundary (e.g., PW, PVs, mitral annulus) and progressively moving away from it. This strategy was adopted to effectively eliminate EGMs and prevent development of iatrogenic slow-conducting sites which could increase the risk of macroreentrant atrial tachyarrhythmias [3,4].

Hospital Management and Follow-up.

All patients were discharged after overnight observation if no peri-procedural complications occurred. AAD therapy was resumed before hospital discharge and subsequently discontinued if no recurrences were documented during a 4-week blanking period. Follow-up visits were scheduled in our outpatient clinic at 1 and 3 months, every 3 months for the first year, and then every 6 months and included in-person clinical evaluation, 12-lead ECG, and 7-day Holter monitoring. Additionally, patients were instructed to contact our staff in case of

novel onset of arrhythmic symptoms potentially requiring an unscheduled in-person assessment.

Transthoracic echocardiography (TTE) was performed before discharge and at approximately 6-9 months, aiming at assessing LA mechanical contraction (A mitral wave).

Supplemental Results.

Echocardiographic Assessments

Transesophageal echocardiography at the end of the procedure did not show any evidence of LAA mechanical activity (late diastolic emptying velocity) in 2 (2.8%) patients; electrical isolation in these patients was confirmed by advancing the PFA catheter in flower configuration onto the LAA ostium.

A subgroup of 36 patients completed a follow-up TTE study to assess LA mechanical contraction changes after ablation (Supplemental Figure 1). Median A-wave velocity was 30 (IQR: 20-35) cm/s post-ablation and 50 (IQR: 41-70) cm/s after a mean of 7.3±1.8 months post-ablation.

Supplemental References.

1. Seitz J, Bars C, Théodore G, et al.: AF Ablation Guided by Spatiotemporal Electrogram Dispersion Without Pulmonary Vein Isolation. J Am Coll Cardiol 2017; 69:303–321.

2. Li K, Xu C, Zhu X, et al.: Multi-centre, prospective randomized comparison of three different substrate ablation strategies for persistent atrial fibrillation. EP Eur 2023; :euad090.

3. Feola I, Volkers L, Majumder R, et al.: Localized Optogenetic Targeting of Rotors in Atrial Cardiomyocyte Monolayers. Circ Arrhythm Electrophysiol 2017; 10:e005591.

4. Shi R, Chen Z, Pope MTB, et al.: Individualized ablation strategy to treat persistent atrial fibrillation: Core-to-boundary approach guided by charge-density mapping. Heart Rhythm 2021; 18:862–870.

Supplemental Figure 1. A wave velocities post-ablation (Red Box) and at FU (Green Box). The band inside the box represents the median. The ends of the box are the first and third quartiles. The whiskers represent the maximum and minimum of all data. FU: follow-up.

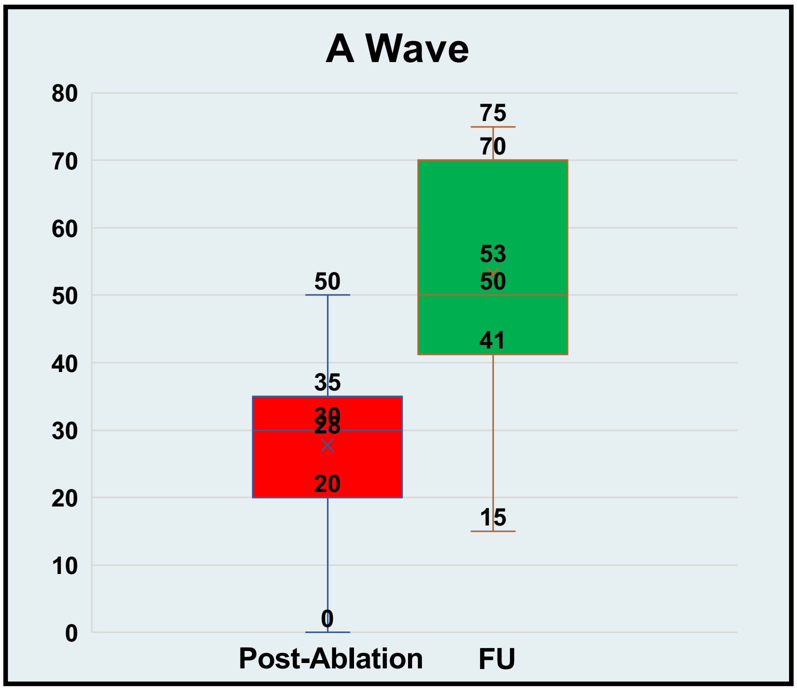

Supplement: euae246_Supplementary_Data [file euae246_supplementary_data.pdf]
